# Coding-Complete Genome Sequence of Staphylococcus aureus Podophage Portland

**DOI:** 10.1128/MRA.01337-19

**Published:** 2019-11-21

**Authors:** Rachele M. Bonasera, Abby Korn, Heather Newkirk, Chandler O’Leary, Jason Gill, Mei Liu

**Affiliations:** a Center for Phage Technology, Texas A&M University, College Station, Texas, USA; DOE Joint Genome Institute

## Abstract

Staphylococcus aureus bacteria, especially the multidrug resistance strains, are responsible for a wide range of clinical infections. Here, we announce the genome sequence of S. aureus podophage Portland, which is closely related to a group of phi29‐like S. aureus podophages, including phages phi44AHJD and phiP68. The exact genome sequence ends of phage Portland were not determined and may be obscured by terminal proteins.

## ANNOUNCEMENT

Staphylococcus aureus is a major human pathogen responsible for a wide range of clinical infections, including pneumonia, bacteremia, hospital-acquired wound infections, and medical device-associated infections (1). The use of different types of antibiotics over the years has led to the emergence of methicillin-resistant S. aureus (MRSA) strains that are often resistant to other classes of antibiotics (2). Given the limited antibiotic treatment options, S. aureus phages may have clinical promise as therapeutic agents for the treatment of S. aureus infections (3).

Phage Portland was isolated from environmental samples collected from a swine barn in Kansas in 2015 against S. aureus strain NRS253. Host bacteria were cultured on tryptic soy broth or agar (Difco) at 30°C with aeration. Phages were cultured and propagated using the soft agar overlay method (4). Portland was identified as a podophage using negative-stain transmission electron microscopy performed at the Texas A&M University Microscopy and Imaging Center, as described previously (5). Phage genomic DNA was prepared using a modified Promega Wizard DNA cleanup kit protocol (5). Pooled indexed DNA libraries were prepared using the Illumina TruSeq Nano low-throughput (LT) kit, and the sequence was obtained from the Illumina MiSeq platform using the MiSeq V2 500-cycle reagent kit, following the manufacturer’s instructions, producing 597,167 paired-end 250-bp reads for the index containing the phage Portland genome. FastQC 0.11.5 (https://www.bioinformatics.babraham.ac.uk/projects/fastqc/) was used to quality control the reads. The reads were trimmed with the FASTX-Toolkit 0.0.14 (http://hannonlab.cshl.edu/fastx_toolkit/download.html) before being assembled using SPAdes 3.5.0 (6). Glimmer 3.0 (7) and MetaGeneAnnotator 1.0 (8) were used to predict protein-coding genes, with manual verification, and tRNA genes were predicted using ARAGORN 2.36 (9). Rho-independent termination sites were identified via TransTermHP (http://transterm.cbcb.umd.edu/). Sequence similarity searches were done using BLASTp 2.2.28 (10), with a maximum expectation cutoff of 0.001 against the NCBI nonredundant (nr), UniProt Swiss-Prot (11), and TrEMBL databases. InterProScan 5.15-54.0 (12), LipoP (13), and TMHMM v2.0 (14) were used to predict protein functions. All analyses were conducted at default settings via the CPT Galaxy (15) and Web Apollo (16) interfaces (https://cpt.tamu.edu/galaxy-pub).

Phage Portland was assembled at 78.6-fold coverage into a contig of 17,711 bp. Portland is closely related to a group of phi29‐like S. aureus podophages (17, 18), within which phages phi44AHJD and phiP68 have been characterized (19). Portland possesses a low G+C content (29.6%) similar to those of its host (32.7%) (20) and to this group of phages (19). As determined using BLASTn against the NCBI nucleotide database, phage Portland shares 89% and 83% nucleotide similarity with S. aureus phages phiP68 (GenBank accession number AF513033) and phi44AHJD (GenBank accession number AF513032), respectively. All 19 predicted proteins in Portland share homology with phage phiP68 (BLASTp; E value, <10^−29^). Direct amplification using primers facing off the Portland genome ends failed to generate a PCR product, consistent with the presence of covalently linked terminal proteins described in this phage group (19, 21, 22). The Portland genome reported in this study is likely missing short sequences from each contig end. Experiments were not conducted to determine the extreme terminal sequences of the Portland chromosome, which may be obscured by terminal proteins.

### Data availability.

The genome sequence of phage Portland was submitted to GenBank as accession number MN098325. The associated BioProject, SRA, and BioSample accession numbers are PRJNA222858, SRR8761742, and SAMN11191518, respectively.
